# Screening for or diagnosing medial meniscal root injury using peripheral medial joint space width ratio in plain radiographs

**DOI:** 10.1038/s41598-023-31735-0

**Published:** 2023-03-27

**Authors:** Pasin Asawatreratanakul, Tanarat Boonriong, Wachiraphan Parinyakhup, Chaiwat Chuaychoosakoon

**Affiliations:** Department of Orthopedics, Faculty of Medicine, Prince of Songkla University, 15 Karnjanavanich Road, Hat Yai, Songkhla 90110 Thailand

**Keywords:** Disability, Clinical trials

## Abstract

To evaluate the sensitivity and specificity for screening and diagnosis of medial meniscal root injury using the distance ratio of medial joint space width between affected and unaffected knees in patients with potential medial meniscal root injury (MMRI) using plain radiographs, the study enrolled 49 patients with suspected MMRI who were then evaluated for MMRI using plain radiographs of both knees in the anteroposterior view and magnetic resonance imaging (MRI) findings. The ratios of peripheral medial joint space width between the affected and unaffected sides were calculated. The cut point value, sensitivity and specificity were calculated according to a receiver operating characteristic (ROC) curve. In the study, 18 and 31 patients were diagnosed with and without MMRI, respectively. The mean peripheral medial joint space width ratios comparing the affected side to the unaffected side in the standing position of the anteroposterior view of both knees in the MMRI and non-MMRI groups were 0.83 ± 0.11 and 1.04 ± 0.16, respectively, which was a significant difference (p-value < 0.001). The cut point value of the peripheral medial joint space width ratio between the affected and unaffected sides for suspected MMRI was 0.985, with sensitivity and specificity of 0.83 and 0.81, respectively, and for diagnosis was 0.78, with sensitivity and specificity of 0.39 and 1.00, respectively. The area under the ROC curve was 0.881. Patients with a possible MMRI had peripheral medial joint space width ratios less than patients with non-MMRI. This test can be used for reliably screening for or diagnosing medial meniscal root injury in primary or secondary care settings.

## Introduction

The incidence of medial meniscal root tear has substantially increased in recent years^1–4^. A medial meniscal root injury is biomechanically equivalent to having no meniscal tissue post-traumatic injury or post-operative total meniscectomy^5^, a condition which can rapidly progress to osteoarthritis if the diagnosis and treatment are delayed^6–9^. Choi et al.^6^ reported that medial meniscal root injury was found in 78.17% of patients less than 60 years old who underwent total knee arthroplasty. In contrast, patients who had been diagnosed at an early stage and treated with meniscal root repair had significantly better Lysholm and International Knee Documentation Committee (IKDC) scores and lower incidences of osteoarthritis^8^.

The clinical symptoms of patients with medial meniscal root injury generally involve a history of minor trauma (i.e. from squatting, changing position from sitting in a hyperflexion knee position to standing position, a low velocity fall, etc.) and persistent medial side knee pain^10,11^, which is usually diagnosed as osteoarthritis even in patients less than 50 years old^12^. To confirm the diagnosis and reduce the incidence of delayed or missed diagnosis of medial meniscal root injury, magnetic resonance imaging (MRI) is the gold standard investigation^13–16^. However, most patients with medial side knee pain first visit a doctor at a primary or secondary care unit where MRI is not available, and it would be beneficial for these patients if this kind of injury could be screened for at a primary or secondary care unit where plain radiographs are a standard investigation and readily available. Two studies, Bloecker et al.^17^ and Gale et al.^18^, evaluated the relationship between plain radiographs and intraarticular pathology using MRI, and both found that the center and periphery of the medial joint space were related to cartilage and meniscus, respectively^17,18^.

There are currently no screening tools readily available at primary or secondary care settings for screening or diagnosing medial meniscal root injury, which can lead to under-diagnosis of this disease with serious consequences for the patients. We felt that a careful examination of certain features of plain radiographs, available in some primary and all secondary care settings, might be able to provide sufficient evidence to indicate a risk of this type of injury, at which time at-risk patients could be referred in a timely way to a higher institute for proper management. The purpose of this study was to evaluate the sensitivity and specificity for screening or diagnosing medial meniscal root injury using the distance ratio of peripheral medial joint space width between affected and unaffected knees using plain radiographs. The hypothesis was that a patient with a medial meniscal root injury would have a smaller peripheral medial joint space width ratio than a patient without a medial meniscal root injury.

## Results

### Study population

Of the 75 patients enrolled, 26 patients were excluded, leaving 49 patients in the study, 18 patients with medial meniscal root injury and 31 patients with non-medial meniscal root injury. The demographic data are shown in Table 1; there were no significant differences between the groups. In the non-medial meniscal root injury group, the pathologies were meniscal tear of the anterior horn, body, or posterior horn in 1, 9, and 9 of 31 patients, respectively. The specific tear types were a degenerative tear in the 1 anterior horn patient, 5 horizontal tears and 4 degenerative tears in the 9 meniscus body tears, and 2 longitudinal tears, 4 horizontal tears and 3 degenerative tears in the 9 posterior horn tears.

**Table 1:** Patient characteristics compared between the medial meniscal root injury and non-medial meniscal root injury groups.

Characteristic	Medial meniscal root injury (18)	Non-medial meniscal root injury (31)	p-value
Age, years (SD)	47.1 (10.2)	42.5 (9.7)	0.125
Gender			0.585
Male	10 (55.6)	21 (67.7)	
Female	8 (44.4)	10 (32.3)	
Body mass index, kg/m^2^ (SD)	25.8 (2.9)	24.5 (3.4)	0.178
Side			0.898
Right	11 (61.1)	17 (54.8)	
Left	7 (38.9)	14 (45.2)	
Cartilage injury: medial compartment			0.783
Grade 0	10 (55.6)	16 (51.6)	
Grade 1	0 (0)	2 (6.5)	
Grade 2	8 (44.4)	13 (41.9)	

The mean medial joint space widths of the affected and unaffected sides of the medial meniscal root injury group were 4.33 ± 1.57 and 5.25 ± 1.60 mm, respectively (p-value = 0.001), and of the non-medial meniscal root injury group were 5.37 ± 1.18 and 5.21 ± 1.16, respectively (p-value = 0.220). The differences in widths between the affected and unaffected sides in the medial meniscal root injury and the non-medial meniscal root injury groups were −0.93 ± 0.99 and 0.16 ± 0.72, respectively (p-value < 0.001).

The mean peripheral medial joint space width ratios comparing the affected side to the unaffected side in the standing position of the anteroposterior view of both knees in the medial meniscal root injury and non-medial meniscal root injury groups were 0.83 ± 0.11 and 1.04 ± 0.16, respectively, which was a significant difference (p-value < 0.001). The agreements between the peripheral medial joint space width ratio of the affected and unaffected sides and MRI findings, with sensitivity and specificity values, are shown in Table 2.

**Table 2:** Overall values for screening parameters (PPV, positive predictive value; NPV, negative predictive value; LR+, likelihood ratio for a positive test; LR–, likelihood ratio for a negative test).

Diagnostic parameters (95% confidence intervals)	
Sensitivity	0.83 (0.58, 0.96)
Specificity	0.81 (0.63, 0.93)
PPV	0.71 (0.48, 0.89)
NPV	0.89 (0.72, 0.98)
LR+	4.31 (2.04, 9.09)
LR−	0.20 (0.07, 0.59)
False positive	0.19 (0.07, 0.37)
False negative	0.17 (0.04, 0.41)

### The cut point value of the peripheral medial joint space width ratio between the affected and unaffected sides for screening for medial meniscal root injury

**Figure 1:**
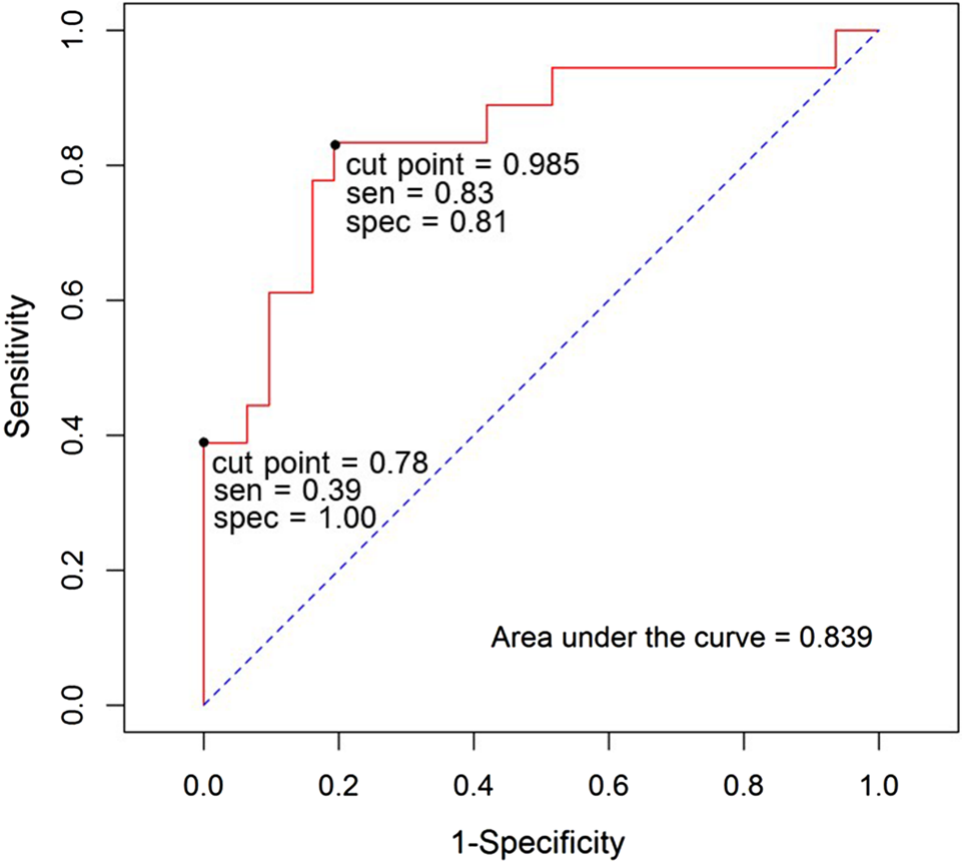
The ROC curve for the basic model with and without medial meniscal root injury.

We used the peripheral medial joint space width ratio between the affected and unaffected sides to discriminate between possible medial meniscal root injury and non-medial meniscal root injury. The cut point value of the peripheral medial joint space width ratio between the affected and unaffected sides for screening for medial meniscal root injury was 0.985; the overall parameters are shown in Table 2. The area under the receiver operating characteristic (ROC) curve was 0.839 (Fig. 1). The power analysis using the cut point value of 0.985 was 0.998.

### The cut point value of the peripheral medial joint space width ratio between the affected and unaffected sides for diagnosing medial meniscal root injury

We used the peripheral medial joint space width ratio between the affected and unaffected sides to discriminate between medial meniscal root injury and non-medial meniscal root injury. The cut point value of the peripheral medial joint space width ratio between the affected and unaffected sides for diagnosing medial meniscal root injury was 0.78; the overall parameters are shown in Table 3. The area under the ROC curve was 0.839 (Fig. 1). The power analysis using the cut point value of 0.78 was 1.

**Table 3:** Overall values for diagnostic parameters (PPV, positive predictive value; NPV, negative predictive value; LR+, likelihood ratio for a positive test; LR−, likelihood ratio for a negative test).

Diagnostic parameters (95% confidence intervals)	
Sensitivity	0.39 (0.17, 0.64)
Specificity	1.00 (0.89, 1.00)
PPV	1.00 (0.59, 1.00)
NPV	0.74 (0.58, 0.86)
LR+	Inf
LR−	0.61 (0.42, 0.88)
False positive	0.00 (0.00, 0.11)
False negative	0.61 (0.42, 0.88)

There was high interobserver correlation between the experienced orthopaedist and the fourth-year orthopaedic resident in all measurements. The correlation coefficients of all assessments ranged between 0.80 and 0.87.

## Discussion

We found that medial meniscal root injuries could be successfully screened for and/or diagnosed using plain radiographs. We found that all patients with a medial meniscal root injury had a smaller peripheral medial joint space width ratio than patients without medial meniscal root injury. The cut point value of the peripheral medial joint space width ratio between the affected and unaffected sides for screening for medial meniscal root injury was 0.985, with sensitivity and specificity of 0.83 (0.58, 0.96) and 0.81 (0.63, 0.93), respectively, and for diagnosing medial meniscal root injury was 0.78, with sensitivity and specificity of 0.39 (0.17, 0.64) and 1.00 (0.89, 1.00), respectively.

Osteoarthritis is an age-related disease which usually occurs in adults over 60 years of age^19^, although it can occur in patients aged less than 60 years following various traumas such as an intraarticular fracture, meniscal injury or osteochondral injury. Choi et al. reported that 78.17% of patients aged less than 60 years who had symptomatic osteoarthritis of the knees and underwent a TKA were found to have a medial meniscal root injury intraoperatively^6^. When this disease occurs in non-elderly patients, it often results from a missed medial meniscal root injury diagnosis, which is of concern since when a medial meniscal root injury is missed, the patient can rapidly develop symptomatic osteoarthritis which is much more difficult to treat and usually has lifelong consequences. The diagnostic gold standard investigation for medial meniscal root injury is MRI^13–16,20^, but this investigation is very expensive and available only in higher level hospitals. It is hard to identify patients at risk for this injury in primary or secondary care units which provide the first level of health care to individuals and families in most communities. It would be very useful if there was a simple investigation that could be used to screen for medial meniscal root injury in these clinics, which could decrease the incidence of delayed or missed diagnosis of this disease.

Two studies have evaluated the relationship between the medial joint space and the intraarticular structure by comparing MRI images and plain radiographs^17,18^. Both studies found that the center of the medial joint space was related to the cartilage and the periphery of the medial joint space was related to the meniscus. We applied the information from these studies to evaluate medial meniscal root injury by measuring the peripheral medial joint space width. Another factor is that if there is a load transfer to the knee joint in patients with medial meniscal root injury, there will be no hoop stress to decrease the peripheral medial joint space width. In this study, we measured the peripheral area of the medial joint space using radiographs taken with the knee in a standing, weight-bearing position to determine if such radiographs could be used for reliable screening or diagnosis of a medial meniscal root injury. We found that when the peripheral medial joint space width ratio between the affected and unaffected knees in patients with medial meniscal root injury was less than 0.985, the sensitivity and specificity were 0.83 and 0.81, respectively, and when the ratio was less than 0.78, the sensitivity and specificity were 0.39 and 1.00, respectively.

Based on the results of this study, we recommend that patients with symptoms indicating possible medial meniscal root injury should have anteroposterior radiographs of both knees with the patient in the standing, weight-bearing position, to perform our test described in this paper to avoid delayed or missed diagnosis of medial meniscal root injury, a disease which can quickly progress to osteoarthritis. These measurements involve quite small distances, so to ensure adequate accuracy when doing the measurements, we recommend first enlarging the image. If the peripheral medial joint space width ratio between the knees is less than 0.985, the doctor should be concerned about medial meniscal root injury (sensitivity = 0.83) and consider referring the patient for further investigation and treatment at a higher-level hospital. A peripheral medial joint space width ratio between the knees of less than 0.78 was strongly associated with medial meniscal root injury (specificity = 1.00).

The primary strength of this study is our confirmation that using a plain radiograph in an anteroposterior standing view of both knees, a simple investigation available in some primary care units and all secondary care units, can be used to screen for or diagnose medial meniscal root injury. This position is achievable in patients with any degree of knee pain, in contrast to the Rosenberg^21^ or one leg standing views^22^. The Rosenberg view is a posteroanterior view with weight-bearing in 45 degrees of knee flexion, while the one leg standing position is an anteroposterior view with full extension and weight-bearing. Either of these positions is hard to maintain for patients with moderate to severe knee pain. Additionally, the measurement method used in our study had high validity and was easy to perform based on clear anatomical landmarks.

There were some limitations to this study. First, it was hard to control the quality of the radiographic images due to the varying knee positions of the patients and the beam tilt, however, we compensated for this problem by using the distance ratio between the affected and unaffected sides. Second, pathology in a knee can lead to a patient not having full extension on an injured knee, leading to altered values even when comparing contralateral knees as each knee may not be at the same angle. Third, there were only small numbers of patients in both groups of this study. For further research, we plan to increase the number of patients through a multicenter trial. Fourth, patients with knee pathologies other than medial meniscal root injury such as modified Outerbridge grade 3 and 4 chondral lesions were excluded, as these pathologies can interfere with the joint space in one or both knees. Fifth, the results from this study would be difficult to apply to a patient with contralateral medial joint space narrowing (i.e. from osteoarthritis), and could thus result in falsely elevated values, and we suggest referring these patients to an orthopedist.

## Conclusion

Patients with a medial meniscal root injury have a peripheral medial joint space width ratio less than patients without medial meniscal root injury. This test can be used for reliably screening for or diagnosing medial meniscal injury in a primary or secondary care settings.

## Material and methods

This prospective review was approved by our institutional review board, and was performed in accordance with the relevant guidelines and regulations (REC 62-276-11-1). 75 patients with medial side knee pain were included in the study, aged between 23 and 69 years, enrolled from December 2019 to November 2021. Informed consent was obtained from all patients. The inclusion criteria were a history of minor trauma within the previous 3 months (i.e. from squatting, changing position from sitting in a hyperflexion knee position to standing position, a low velocity fall, etc.), pain on deep flexion, and tenderness at the medial joint line. The exclusion criteria were cartilage injury grade 3 or greater as shown on MRI (using the modified Outerbridge classification^23^), or a history of knee surgery, rheumatoid disease, soft tissue or bone tumors, or contralateral knee pain.

All patients had MRIs of the affected knee and anteroposterior view plain radiographs of both knees taken in the standing position.

### Joint space width measurements

**Figure 2:**
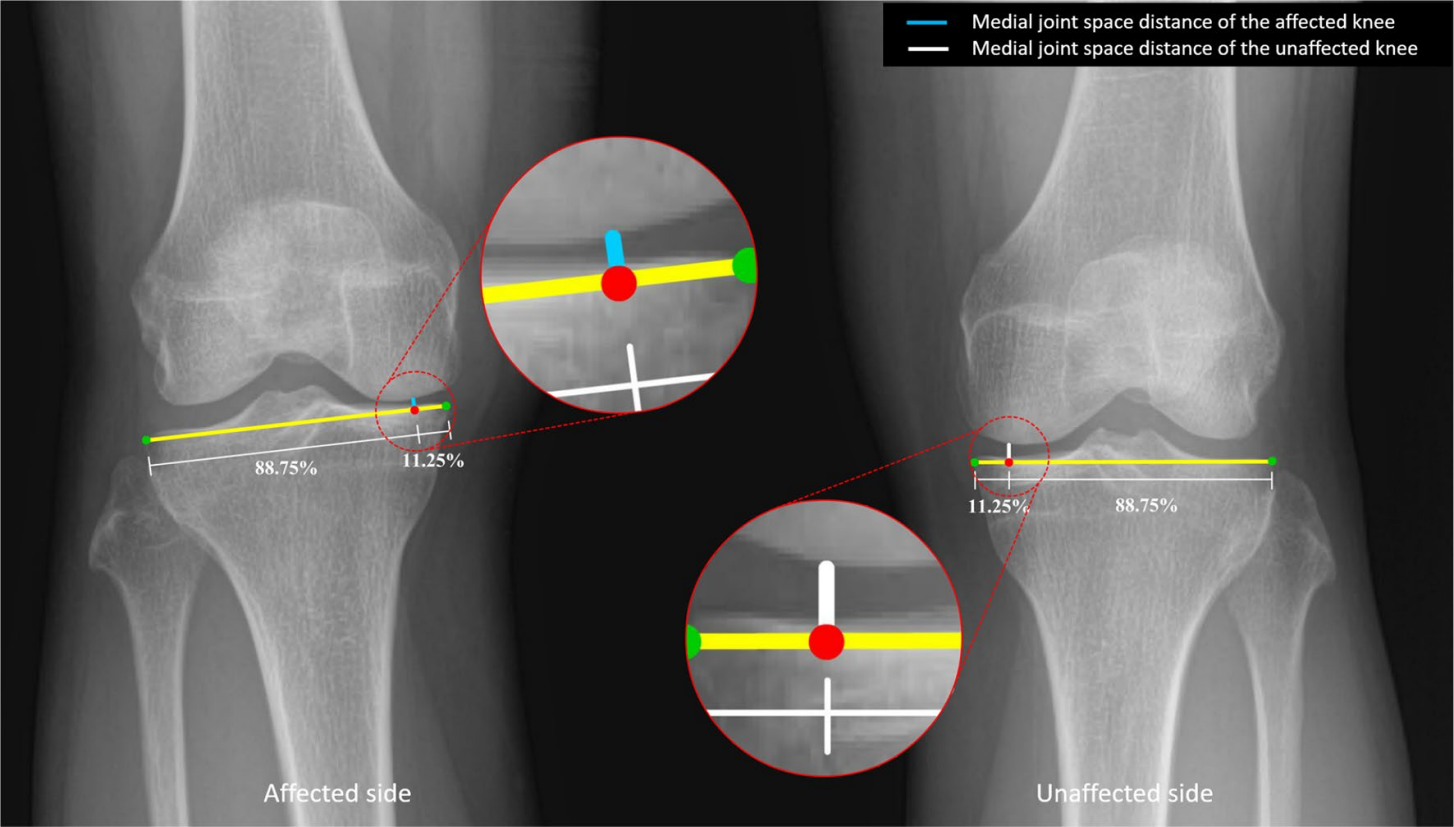
An anteroposterior radiographic image of both knees showing the peripheral medial joint space distance between the medial femoral condyle and the medial tibial plateau of the affected (blue line) and unaffected (white line) knees. The study analysis was based on the ratio of the peripheral medial joint space distance in the affected knee (**A**, blue line) relative to the peripheral medial joint space distance in the unaffected knee (**B**, white line) (**A**, **B**).

Medial joint space width refers to the cartilage and meniscal structure of the knee. Two previous studies have reported that the center of the medial joint space was related to the cartilage and the periphery of the medial joint space was related to the meniscus^17,18^. To measure the joint space width, the distance from the most medial point to the most lateral point (yellow line in Fig. 2) was measured, and 11.25% of this distance measured inward from the most medial point of the tibial plateau was taken as the landmark (Fig. 2, red dot). The peripheral medial joint space width was measured perpendicularly from the red dot on Fig. 2 to the medial femoral condyle (white and blue lines). The ratio was then calculated by dividing the peripheral medial joint space width of the affected knee by the same measurement on the unaffected side. Each peripheral medial joint space width was measured three times by one experienced orthopaedist and one fourth-year orthopaedic resident, both of whom were blinded to the clinical data and MRI results. MRI results were reported by an experienced musculoskeletal radiologist.

### Statistical analysis

The statistical analysis was performed with the R program and epicalc package (version 3.4.3, R Foundation for Statistical Computing, Vienna, Austria). The differences between the medial joint space width ratio of the affected and unaffected sides were analyzed using unpaired t-test. Statistical significance was set at P ≤ 0.05. The results are presented as mean ± SD. The parameters for diagnosing medial meniscal root injury of the medial joint space width ratio of the affected and unaffected sides were calculated in relation to the MRI findings, with sensitivity and specificity calculated for a 95% CI, and positive and negative predictive values. Inter-observer and intra-observer reliabilities were assessed by calculating intraclass correlation coefficient values (Supplementary file).

### Supplementary Information

Supplementary Information.

## Data Availability

The datasets generated and/or analysed during the current study are available from the corresponding author on reasonable request.
