# Author Correction: Screening for or diagnosing medial meniscal root injury using peripheral medial joint space width ratio in plain radiographs

**DOI:** 10.1038/s41598-023-47677-6

**Published:** 2023-11-22

**Authors:** Pasin Asawatreratanakul, Tanarat Boonriong, Wachiraphan Parinyakhup, Chaiwat Chuaychoosakoon

**Affiliations:** Department of Orthopedics, Faculty of Medicine, Prince of Songkla University, 15 Karnjanavanich Road, Hat Yai, Songkhla, 90110 Thailand

Correction to: *Scientific Reports* 10.1038/s41598-023-31735-0, published online 27 March 2023

The original version of this Article contained an error in the Material and methods section, where the patient inclusion period was incorrect.

“75 patients with medial side knee pain were included in the study, aged between 23 and 69 years, enrolled from December 2020 to November 2021.”

now reads:

“75 patients with medial side knee pain were included in the study, aged between 23 and 69 years, enrolled from December 2019 to November 2021.”

The original Article has been corrected.

